# Gene Expression Variation in Duplicate *Lactate dehydrogenase* Genes: Do Ecological Species Show Distinct Responses?

**DOI:** 10.1371/journal.pone.0103964

**Published:** 2014-07-31

**Authors:** Melania E. Cristescu, Bora Demiri, Ianina Altshuler, Teresa J. Crease

**Affiliations:** 1 Great Lakes Institute for Environmental Research, University of Windsor, Windsor, Ontario, Canada; 2 Department of Integrative Biology, University of Guelph, Guelph, Ontario, Canada; 3 Department of Biology, McGill University, Montreal, Quebec, Canada; Consiglio Nazionale delle Ricerche (CNR), Italy

## Abstract

Lactate dehydrogenase (LDH) has been shown to play an important role in adaptation of several aquatic species to different habitats. The genomes of *Daphnia pulex*, a pond species, and *Daphnia pulicaria*, a lake inhabitant, encode two L-LDH enzymes, LDHA and LDHB. We estimated relative levels of *Ldh* gene expression in these two closely related species and their hybrids in four environmental settings, each characterized by one of two temperatures (10°C or 20°C), and one of two concentrations of dissolved oxygen (DO; 6.5–7 mg/l or 2–3 mg/l). We found that levels of *Ldh*A expression were 4 to 48 times higher than *Ldh*B expression (p<0.005) in all three groups (the two parental species and hybrids). Moreover, levels of *LdhB* expression differed significantly (p<0.05) between *D. pulex* and *D. pulicaria,* but neither species differed from the hybrid. Consistently higher expression of *Ldh*A relative to *Ldh*B in both species and the hybrid suggests that the two isozymes could be performing different functions. No significant differences in levels of gene expression were observed among the four combinations of temperature and dissolved oxygen (p>0.1). Given that *Daphnia* dwell in environments characterized by fluctuating conditions with long periods of low dissolved oxygen concentration, we suggest that these species could employ regulated metabolic depression to survive in such environments.

## Introduction

Identifying the molecular changes that contribute to the adaptation of a species to its local environment is an important goal of evolutionary studies [1]. Adaptation to a new environment may be accomplished by molecular changes in enzymes that alter the intrinsic enzymatic rate constants, by a heritable change in the control region of the genome influencing transcript level and consequently enzyme concentration, or by epigenetic factors [2]. Studies investigating the genetic basis of fitness differences in natural populations are often based on protein coding genes involved with glucose metabolism [3], [4], [5], [6]. One such group of enzymes is the NAD-dependent L-lactate dehydrogenases (LDH), which are required by most organisms to reduce pyruvate to L(−) lactate during glycolysis and oxidize L(−) lactate to pyruvate during gluconeogenesis [7].

LDH has been shown to contribute to the adaptation of natural populations in different habitats [2], [8], [9], [10], [11]. For instance, populations of *Fundulus heteroclitus* in Maine and Georgia are fixed for two different alleles at the heart-type *Ldh*B locus. The isozymes encoded by this locus have different temperature-dependent kinetic characteristics due to a few amino acid replacements making these two populations more adapted to their different thermal environments [8]. In addition, an increase in the activity of LDH in the killifish, *F. heteroclitus,* is observed during prolonged exposure to hypoxia [12] indicating the importance of this enzyme in the adaption to different environmental conditions.

A strong association between *Ldh* genotype and habitat has also been observed in members of the *Daphnia pulex* species complex. The pond species, *D. pulex* and the closely related lake species, *D. pulicaria* are fixed for different alleles at the *Ldh*A locus. *Daphnia* in ephemeral ponds are homozygous for the *Ldh*A slow allele (S), whereas *Daphnia* in permanent lakes are homozygous for the *Ldh*A fast allele (F), where S and F refer to relative mobility through gels during protein electrophoresis [13], [14], [15], [16]. Hybrids of the two species, with the heterozygous SF (Slow/Fast) genotype at the *Ldh*A locus, can be found in disturbed, deforested ponds [17] or intermediate habitats. Detailed phylogenetic studies revealed that the main differences between the S and F alleles of the *Ldh*A locus are amino acid substitutions at two sites [18]. This includes the substitution of Aspartic acid in the S allele for Glutamic acid in the F allele at amino acid position 6, and the substitution of Glutamine in S for Glutamic acid in F at position 229, which likely causes the slow/fast mobility shift. Considerably more amino acid variation was observed among LDHB proteins [18].

Several studies have found that *Daphnia* in lakes and ponds differ considerably in their life history traits [19], [20], [21] since these habitats are characterized by different physical and biotic conditions [22]. *Daphnia pulex* populations inhabit fishless, ephemeral ponds for a short period of time in the spring and early summer where they are exposed to anoxia, and eventually desiccation or complete freezing [23]. Conversely, *D. pulicaria* populations can be found in the cold hypolimnetic region of stratified lakes in order to avoid fish predation [24]. It is likely that these two species have diverged due to distinct environmental conditions in their specific habitats such as temperature, dissolved oxygen concentration, biotic interactions, and food sources. However, despite advances in understanding the ecology of *Daphnia* and the availability of new genomic tools, we lack an understanding of the molecular mechanisms used by these species to adapt to ephemeral pond versus permanent lake habitats.

In addition to LDHA, the genomes of *D. pulicaria* and *D. pulex* encode a more genetically variable, second isoenzyme, LDHB [25]. Based on phylogenetic studies, the two loci (*Ldh*A and *Ldh*B) seem to be the product of a relatively recent gene duplication event in *Daphnia*, after its divergence from other crustaceans [25]. The fixation of different *Ldh*A alleles in these two sister species, which occur in different aquatic habitats, suggests that LDH variation could be adaptive, or *LdhA* is linked to loci that are under selection [14], [25]. Therefore, it is of interest to estimate variation in gene expression among these sister species in controlled laboratory conditions.

This study determines the expression patterns of both isoenzymes, *Ldh*A and *Ldh*B, in clones of *D. pulicaria*, *D. pulex* and *D. pulex/pulicaria* hybrids that have been acclimatized to four different environmental settings. Each setting was characterized by one of two temperatures (10°C or 20°C), and one of two concentrations of dissolved oxygen (DO, 6.5–7 mg/l or 2–3 mg/l).

## Materials and Methods

### Sample collection

This study involved collection of *Daphnia* from ponds and lakes. No specific permissions are required to sample *Daphnia* as they are not endangered or protected species. Lakes were accessed via public boat ramps while ponds were sampled from public roadsides or on private land with the permission of the land owner. A total of eight habitats (three lakes and five ponds) were sampled in Michigan and southern Ontario (Figure S1) during early spring, soon after hatching, when genetic diversity is high [26]. Individual female *Daphnia* were taken to the laboratory and allowed to reproduce parthenogenetically in separate beakers. After genotyping (see below), three genetically unique clonal lines were selected from each of the three pond populations of *D. pulex* (Canard 3E, Solomon, Disputed), and each of the three lake populations of *D. pulicaria* (Warner, Three Lakes II, Lawrence) giving nine clones per species. In addition, we isolated nine *D. pulex/pulicaria* hybrid lines. Four lines were established from pond Canard 1B, three lines were established from pond Canard 3L and one line was established from each of ponds Canard 3 and Disputed. The isolates were maintained in filtered river water at 15−18°C with a 14-h light, 10-h dark photoperiod and fed every 3–4 days with a combination of the microalgae species *Nannochloropsis* and *Tetraselmis* (Reed Mariculture).

### Genotyping and sexuality tests

Isolates from both pond and lake populations were initially genotyped at 21 previously mapped microsatellite markers by Cristescu et al. [27] and Xu et al. [28]. The 12 *D. pulex* linkage groups were each represented by one to three loci [29]. Isolates with unique multilocus genotypes were selected for the gene expression experiment. In order to identify *D. pulex/pulicaria* hybrids from the pond populations, the *Ldh*A genotype of each isolate was also determined by Cristescu et al. [27] and Xu et al. [28]. Previous surveys of allozyme variation have shown that lake populations are fixed for an electrophoretically “fast” (F) allele at the *Ldh*A locus [26], [30] while pond populations are either fixed for the “slow” (S) allele or contain SF heterozygotes. The heterozygotes are considered to be hybrids of *D. pulex* and *D. pulicaria* [15], [28] and are reported to reproduce by obligate parthenogenesis [26]. We used S and F allele-specific primers based on *Ldh*A sequences from both species [25] to determine the genotype of each isolate. Sexuality tests were conducted on the pond isolates to determine their mode of reproduction (cyclical parthenogenesis or obligate parthenogenesis), using the method of Innes *et al*. [26], which involves rearing female *Daphnia* in the absence of males and observing their ability, or lack thereof, to deposit eggs (dormant embryos) into their ephippial egg case.

### Acclimation and experimental methods

The 27 clonal lines were cultured under standardized conditions, as described above, for a period of one year to eliminate maternal effects. Progenitor females from each line were selected to establish the mother generation of the acclimation experiments.

**Figure 1:**
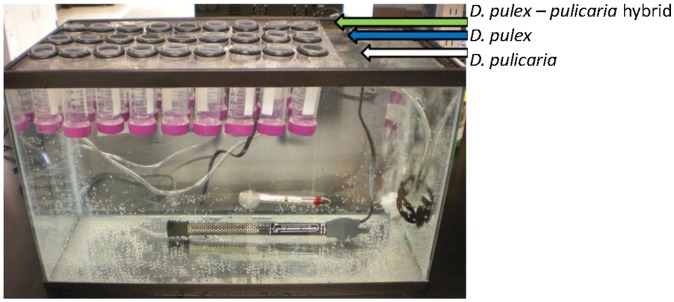
Aquarium set up. Each of the four aquaria contained 27 chambers that were filled with one of nine *D. pulex* clones (pond), nine *D. pulicaria* clones (lake), and nine hybrid clones. Each chamber had a mesh bottom allowing food and air/nitrogen to circulate freely within the aquarium while keeping the clones isolated from each other. There were four aquaria each characterized by different environmental conditions. Aquarium 1 corresponds to 20°C/High [6.5–7 mg/L] DO; aquarium 2 (20°C/Low [2–3 mg/L] DO); aquarium 3 (10°C/High DO); and aquarium 4 (10°C/Low DO).

The four treatments were characterized by a combination of two temperatures, 10°C or 20°C, and two concentrations of dissolved oxygen (DO), 6.5–7 mg/l or 2–3 mg/l. The high DO concentration was obtained by bubbling air into the aquaria using a Topfin Airpump 8000, for 30 min twice a day. The low DO concentration was obtained by bubbling nitrogen gas in the same manner. Each of the four aquaria contained 50-ml chambers each inoculated with one juvenile female from each of the 27 genetically distinct clones. The floor of the chambers was 60-micron mesh, which allowed water and food to circulate freely throughout the aquarium and the chambers (Figure 1). This experimental design allowed the maintenance of uniform experimental conditions across the *Daphnia* clones while keeping them physically separated from one another. The *Daphnia* were exposed to a 14 h light: 10 h dark cycle and were fed daily, at libitum with the unicellular, previously frozen, green algae *Scenedesmus* to maintain an equivalent of 1 mg of C/L in the aquaria. The first clutch of offspring produced by each female was removed from the chamber. Once a female produced the 2^nd^ and 3^rd^ clutch, she was removed and the offspring were left in the chamber to mature. When these offspring reached maturity and eggs could be seen in their brood pouches, they were collected, placed in 400 µL of RNA later (Ambion), and frozen instantly in liquid nitrogen to prevent RNA degradation. The samples were then stored at −80°C until total RNA was extracted.

### RNA isolation and cDNA synthesis

Total RNA was extracted from four to six individuals from each chamber using the RNA extraction and purification protocol developed by the *Daphnia* Genomic Consortium [31]. RNA purification was performed using the RNeasy Mini Kit (QIAGEN, Toronto, Canada). During RNA purification the samples were exposed to RNase-free DNAse I for 15 min prior to RNA elution. The purified nucleic acids were re-suspended in 30 µl of RNAse-free water. The concentration of the RNA samples was checked with the NanoVue spectrophotometer (GE Healthcare Technologies) and the ratio of absorbance at 260 nm/280 nm was measured. All samples used for the experiment had a ratio of ∼2.0, which is generally accepted to represent RNA that is contaminated with little or no DNA. PCR reactions lacking Reverse Transcriptase were run on the RNA samples to confirm that there was no DNA contamination.

Forty ng of RNA from each extraction was reverse-transcribed with the Sensiscript Reverse Transcription Kit (QIAGEN) according to the manufacturer’s protocol, using Oligo-dT primers from Ambion at a final concentration of 1 µM, and Sensiscript reverse transcriptase enzyme. The reverse transcription reaction was carried out at 37°C for 60 minutes with 20 µl total volume.

### Quantitative PCR

Primers for quantitative PCR (qPCR) analysis of *Ldh*A and *Ldh*B expression (Table 1) were designed from sequences downloaded from the DOE Joint Genome Institute’s *Daphnia pulex* database using Integrated DNA Technologie’s PrimerQuest^SM^ program, which is based on the Primer3 code [32]. The primer sequences (Table 1) for the reference gene, *Glyceraldehyde-3-phosphate dehydrogenase* (*Gapdh*), were obtained from Spanier et al. [33]. The primers were designed to generate 90–216 base pair (bp) amplicons and the primer pair efficiency ranged from 94.9% to 96.6%. Primer pair efficiency was estimated using LinRegPCR v. 11.0 [34], [35], which determines the mean PCR efficiency of a primer pair by a linear regression fit of the data in the exponential phase of the qPCR reaction. Three qPCR reactions were performed on each RNA sample in a total volume of 20 µl that contained 10 µl of 2X SYBR Premix Ex Taq™ master mix (Takara Bio USA, Madison, WI), 2 µl of cDNA template (made from 40 ng of RNA in 20 µl volume), 0.4 µl of ROX™ Reference Dye, 6.8 µl of H_2_O, and 0.4 µl (10 µM) each of the forward and reverse primers. Negative control reactions without cDNA template were also included to check for non-specific signal due to PCR artifacts such as primer dimerization. Reactions were carried out in clear, qPCR compatible, 96 well plates (Axygen, Waltham, MA). Amplification was performed on an ABI 7500 Real-Time thermal cycler (Applied Biosystems, Foster City, CA) using the thermal cycling parameters recommended for the SYBR Premix Ex Taq™: 95°C for 10 sec, followed by 40 cycles of 95°C for 5 sec and 60°C for 34 sec. The threshold was set to 0.0276. A melting curve analysis was performed at the end of each qPCR reaction. Only one peak was observed, and there were no primer dimers detected in the qPCR reactions.

**Table 1:** Primers used for qPCR analyses of *Ldh* expression in *D. pulex* and *D. pulicaria*.

Gene Code	Gene Name	Function	Gene ID*	Primer Sequences [5′ to 3′]	Amplicon size [bp]	Localization in gene	PCR Efficiency
*Ldh*A	L-Lactate dehydrogenase A	GlycolyticEnzyme	Dappu- 230172	ATCCAGACTCCTGTTGCCCATTCA	216	Forward primer in 2^nd^ exon,reverse in 3^rd^ exon	94.9%
				TTCGCCCTTGAGTTTGTCCTCCAT			
*Ldh*B	L-lactate dehydrogenase B	GlycolyticEnzyme	Dappu- 61140	TTGTCCAATACAGTCCCGACACCA	90	4^th^ exon	96.6%
				GCAACCCACTGAGTTTCCAAGCAA			
*Gapdh*	glyceraldehyde-3-phosphatedehydrogenase	Glycolytic Enzyme	Dappu-302823	TGGGATGAGTCACTGGCATAC	136	3rd exon	95.2%
				GAAAGGACGACCAACAACAAAC			

*The gene ID refers to the *Daphnia pulex* draft genome annotation (dappu v1.1) at www.jgi.doe.gov.

### Data analysis and statistics

The value of 2^−ΔCT^ was used to determine the level of *Ldh*A and *Ldh*B expression relative to *Gapdh* expression, which is assumed to be constant across treatments [36], in each clone in each of the four treatments. Triplicate C_T_ values for each RNA sample were averaged before performing the −ΔC_T_ calculations. A univariate analysis of variance (ANOVA) was done on the ΔC_T_ values using IBM SPSS STATISTICS 19.0.0, to determine significant differences in *Ldh* relative to *Gapdh* gene expression in samples from different treatments. Because the overall comparisons of groups (*D. pulex*, *D. pulicaria*, hybrids) within genes were significant, pairwise group comparisons of the C_T_ values were also done using a t-test with the Bonferroni correction.

## Results

### *Ldh* genotyping and sexuality tests

Our sexuality tests confirmed that the hybrid isolates reproduced by obligate parthenogenesis and the *D. pulex* and *D. pulicaria* isolates reproduced by cyclic parthenogenesis. These results were consistent with the *LdhA* results reported by Cristescu et al. [27] and Xu et al. [28]. Lake isolates (*D. pulicaria*) were fixed for an electrophoretically “fast” (F) allele at the *Lactate dehydrogenase* A *(Ldh*A*)* locus while sexual pond isolates (*D. pulex*) were fixed for a “slow” (S) allele. All SF heterozygote isolates were considered to be hybrids between *D. pulex* and *D. pulicaria*.

### Quantitative PCR

**Figure 2:**
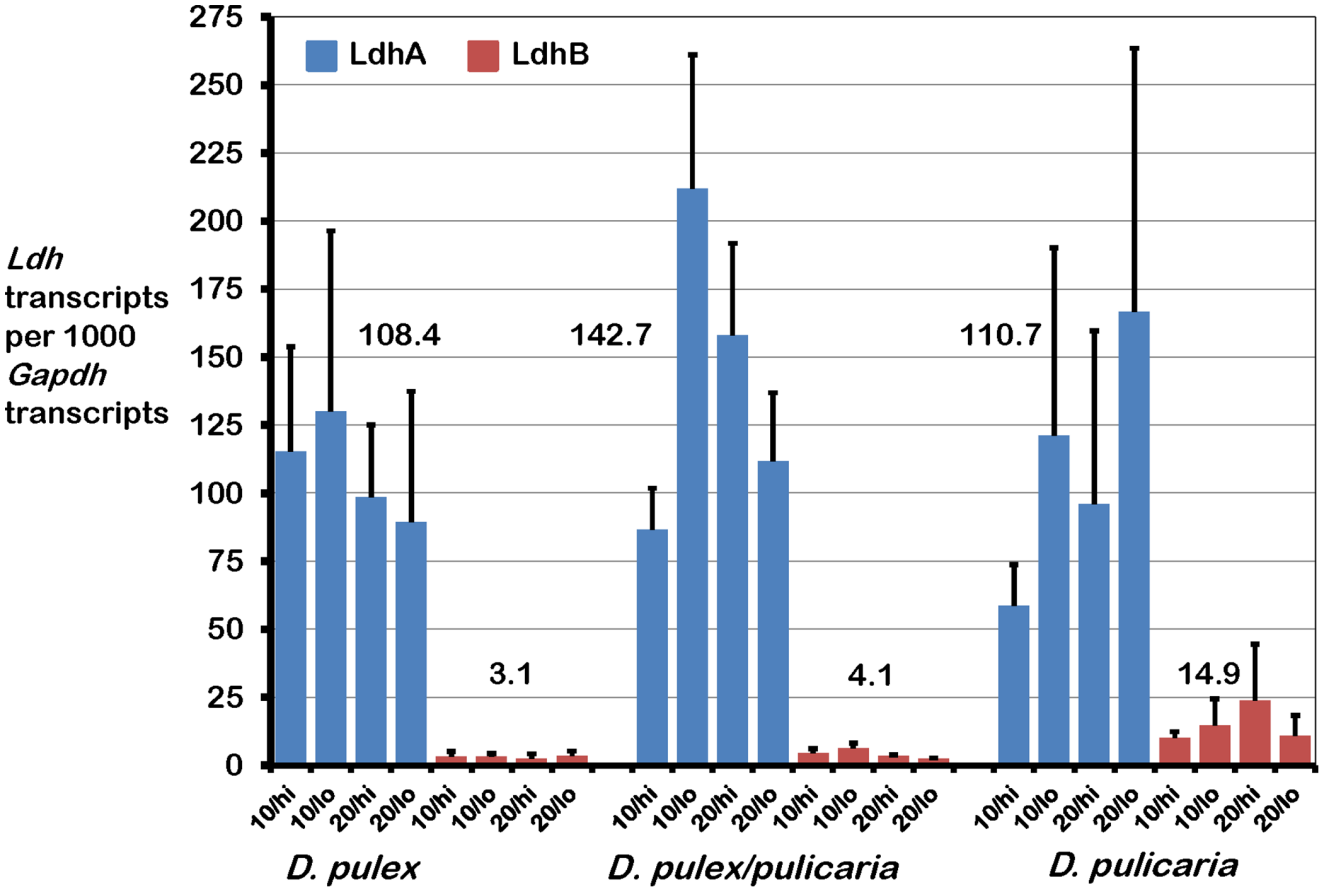
Expression of *Ldh*A and *Ldh*B relative to *Gapdh* in *Daphnia* under four combinations of temperature and dissolved oxygen. Each *Daphnia* group (*D. pulex*, *D. pulicaria* and hybrids) was represented by six clones in the final analysis. The Y-axis shows the number of *Ldh*A and *Ldh*B transcripts per 1000 *Gapdh* transcripts calculated using the 2^−ΔCT^ method. The four treatments consisted of two temperatures, 10°C or 20°C each combined with two levels of dissolved oxygen, 6.5–7 mg/L (hi) or 2–3 mg/L (lo). Blue bars represent *LdhA* and red bars represent *LdhB*. Error bars represent standard error of the mean (SEM). The numbers on the graph are the mean value of relative *Ldh* expression for each treatment in each group. Only the difference between mean relative *Ldh*B expression in *D. pulex* (3.1) and *D. pulicaria* (14.9) is significant (Table 3).

Due to the loss of some clones during the experiments, the analyses are based on the six clones from each of the three groups (for a total of 18 clones) for which we obtained data from all four treatment tanks (Tables S1, S2, S3 in File S1). When more than six clones survived, we selected six clones that maximized the number of shared clones between treatments. The expression of *Ldh*A was significantly different from *Ldh*B in all three groups; *D. pulex, D. pulicaria*, and the hybrid *D. pulex/pulicaria* (source = Gene, Table 2), although both genes were expressed at a much lower level than the reference gene, *Gapdh* (Tables S1, S2, S3 in File S1). In particular, *Ldh*A was expressed 26 to 40 times more than *Ldh*B in *D. pulex*, 4 to 15 times more than *Ldh*B in *D. pulicaria*, and 20 to 48 times more than *Ldh*B in *D. pulex-pulicaria* hybrids (Figure 2). The ANOVA also showed a significant difference in gene expression among the three *Daphnia* groups for each gene (source = Group, Table 2). *Ldh*A expression was similar in *D. pulex* and *D. pulicaria* but higher in the hybrids (Figure 2, Tables S1, S2, S3 in File S1). However, none of the pairwise group differences were significant, although p-values for comparisons between the hybrid and each parent species were less than 0.1 (Table 3). Conversely, *Ldh*B expression was significantly higher in *D. pulicaria* than in *D. pulex* and although expression in the *D. pulex/pulicaria* hybrids was more similar to that in *D. pulex* than *D. pulicaria*, it was not statistically significantly different from either parental species (Figure 2, Table 3, Tables S1, S2, S3 in File S1). Despite significant differences in *Ldh* expression between the two genes and the different groups, there was no significant variation in expression of either gene due to temperature or dissolved oxygen (Table 2).

**Table 2:** Analysis of variance of relative *Ldh* expression in 18 clones of *Daphnia pulex, D. pulicaria* and hybrids.

Gene	Source	Df	F	p-value
	**Gene**			**0.000**
*Ldh*A	Dissolved Oxygen (DO)	1	1.089	0.301
	Temperature (Temp)	1	0.366	0.547
	**Group**	**2**	**3.286**	**0.044**
	DO * Temp	1	0.421	0.519
	DO * Group	2	0.905	0.410
	Temp * Group	2	0.247	0.782
*Ldh*B	Dissolved Oxygen (DO)	1	0.384	0.538
	Temperature (Temp)	1	2.881	0.095
	**Group**	**2**	**9.948**	**0.000**
	DO * Temp	1	0.047	0.829
	DO * Group	2	0.228	0.797
	Temp * Group	1	0.014	0.986

The number of *Ldh* transcripts per *Gapdh* transcript was calculated using the 2^−ΔCT^ method and is the dependent variable in this analysis. Gene is *Ldh*A or *Ldh*B; Group refers to *D. pulex*, *D. pulicaria*, and *D. pulex-pulicaria* hybrids; temperature is 10°C or 20°C; dissolved oxygen is high (6.5–7 mg/l) or low (2–3 mg/l). Interactions are indicated by an asterisk (*). Sources of variation that show a significant difference in gene expression (p<0.05) are in bold-face type.

**Table 3:** Pairwise comparison of *Ldh*A *and Ldh*B expression between genotypes in *Daphnia*.

Gene	Group	Group	p-value
*Ldh*A	*D. pulex*	*D. pulicaria*	1.000
	*D. pulex*	*D. pulex - pulicaria* hybrid	0.093
	*D. pulicaria*	*D. pulex - pulicaria* hybrid	0.088
*Ldh*B	*D. pulex*	*D. pulicaria*	**0.000**
	*D. pulex*	*D. pulex - pulicaria* hybrid	0.056
	*D. pulicaria*	*D. pulex - pulicaria* hybrid	0.139

The results are based on post-hoc t-test with the Bonferroni correction. The number of *Ldh* transcripts per *Gapdh* transcript was calculated using the 2^−ΔCT^ method and is the dependent variable in this analysis. Genotype refers to *D. pulex*, *D. pulicaria*, and *D. pulex-pulicaria* hybrids; Gene is *Ldh*A or *Ldh*B. P-values that show a significant difference in gene expression (p<0.05) are in bold-face type.

## Discussion

Previous studies revealed that the genes encoding the LDHA and LDHB isozymes are under purifying selection with *Ldh*A under stronger evolutionary constraint than *Ldh*B [25]. This suggests that while both isozymes likely produce functional enzymes, *Ldh*A may be more important in *Daphnia* metabolism, which is also consistent with the observation that *Ldh*A expression is significantly higher than *Ldh*B expression in both species and their hybrids, in all four environmental conditions. Substantially higher levels of *Ldh*A than *Ldh*B expression were also observed in a previous study of *Ldh* in these two species under a single set of environmental conditions [37]. The pairwise comparisons show that mean levels of *Ldh*B expression differ significantly between *D. pulicaria* and *D. pulex* with the former group having higher expression of the gene compared to the latter. However, neither of these species was significantly different from the *D. pulex/pulicaria* hybrids, which showed an intermediate level of *LdhB* expression.

It is generally assumed that the duplicate gene is redundant in function immediately after a duplication event. Thus, deleterious mutations in the control region that reduce or eliminate expression of one duplicate may have little effect on overall fitness, and could ultimately drift to fixation [38], [39]. Alternatively, the duplicated genes could undergo subfunctionalization [39] such that they perform only a subset of functions performed by the original protein, and/or the genes are only expressed in specific tissues. Such specialization does occur in jawed vertebrates in which there are three LDH isozymes found predominantly in three different types of tissue; anaerobic tissues (e.g. white skeletal muscle), aerobic tissues (e.g. heart, brain), and spermatozoa of mammals and birds [40]. Our results suggest that LDH specialization could also have occurred in *Daphnia*, such that the relative expression of *Ldh*A and *Ldh*B is correlated with the quantity of tissues in which each is expressed. However, further investigation is needed to confirm this hypothesis. The overall expression of *Ldh*B was extremely low in all three groups, in all four environmental conditions, and further analysis will be required to determine if the mRNA transcripts are actually translated into protein. It is possible that this gene is no longer expressed at a functional level, although analysis of sequences from 85 isolates from 9 different *Daphnia* species suggests that selection is maintaining a functional *Ldh*B protein-coding sequence [18].

LDH catalyzes the final step of the anaerobic metabolic pathway, glycolysis [41] and *Ldh* gene expression has been linked with adaptation of species to environments characterized by different dissolved oxygen concentrations and temperatures. For example, gene expression differences are greater between northern and southern populations of *Fundulus heteroclitus* than between southern *F. heteroclitus* and its sister species *F. grandis* [9]. These patterns of gene expression may be a result of the northern *F. heteroclitus* population adapting to colder water and the southern *F. heteroclitus* and *F. grandis* adapting to warmer water [9]. However, temperature did not affect the expression of either *Ldh* gene in *D. pulex, D. pulicaria*, or the hybrids in our study despite the fact that temperature regimes can be very different in ponds and lakes. For example, shallow ponds may be very cold in the spring when the *Daphnia* first hatch from diapausing eggs, but then warm rapidly throughout the spring and early summer. Conversely, the hypolimnion of deep stratified lakes, where *Daphnia* seek refuge from fish predation during the day [42], can be close to 4°C all year round. Thus, it is possible that the temperature range to which *Daphnia* were exposed in our experiments was not broad enough to elicit a stress response.

In low oxygen conditions, glycolysis rate should be enhanced in order to compensate for oxidative energetic metabolism decay. Moreover, pyruvate to lactate transformation must be efficient in order to keep a high NAD^+^/NADH ratio and therefore allow continued glycolytic flux [41]. In mammals [43] and fish [12], genes encoding proteins involved in the homeostatic response to hypoxia, such as LDH are over-expressed in acute hypoxic conditions. However, Daphnia *Ldh* expression was not found to be up- regulated under low oxygen supply. This could be due to the fact that *Daphnia* is a hypoxia-tolerant invertebrate in which a response to prolonged periods of hypoxia includes regulated metabolic depression [44], [45], which is a stress-coping mechanism that relies on energy conservation and involves the suppression of every major ATP-utilizing function in the cell [45]. *Daphnia pulex* and the hybrids inhabit shallow temporary ponds with fluctuating DO concentrations that range from 5.2 mg/L to 1.1 mg/L. Conversely, *D. pulicaria* inhabits the hypoxic hypolimnion of lakes. Therefore, these *Daphnia* species dwell in environments characterized by long periods of low DO concentration and could be employing regulated metabolic depression to survive in such environments. Further studies are needed to elucidate the mechanism involved in this adaptation.

## Conclusions

We studied whether the expression of the two *Ldh* loci (*Ldh*A and *Ldh*B) varies under different environmental conditions in *D. pulicaria*, *D. pulex* and their hybrids. We observed significant differences in the level of *Ldh*B expression compared to *Ldh*A (p<0.001), with *Ldh*A being expressed 4 to 48 times more than *Ldh*B in all three groups and in all combinations of temperature and oxygen concentration. These results suggest that the function of these two genes might be undergoing specialization such that *Ldh*B is expressed only in a particular set of tissues or organs. Furthermore, although mean *Ldh*A expression was higher in the hybrids, none of the differences between the three *Daphnia* groups were significant. However, mean *Ldh*B expression was significantly higher in *D. pulicaria* than *D. pulex*, and although the level of expression in the hybrid was closer to that in *D. pulex*, it was not significantly different from expression in *D. pulicaria*. Future research on LDHA and LDHB enzyme activity, thermo-stability and rate of protein synthesis under different environmental settings could help clarify the strong association between *Ldh*A genotype and habitat that has been observed in previous studies.

## Supporting Information

Figure S1 Distribution of *Daphnia* sampling sites in Michigan and Ontario. The five ponds are Disputed, Solomon and three ponds in the Canard area. The three lakes are Lawrence, Three Lakes II, and Warner.
(PDF)

File S1 Contains Table S1, CT values for all *Daphnia* clones and qPCR replicates included in this study. Table S2, Gene expression of *Ldh*A and *Ldh*B relative to the reference gene, *Gapdh* in 18 clones of *Daphnia* across four combinations of temperature and dissolved oxygen. Table S3, Gene expression of *Ldh*A and *Ldh*B relative to the reference gene, *Gapdh* in three groups of *Daphnia* clones across four combinations of temperature and dissolved oxygen.
(XLSX)
