# Supplementary material for: Gene Expression Variation in Duplicate Lactate dehydrogenase Genes: Do Ecological Species Show Distinct Responses?
Source: PLoS One. 2014 Jul 31;9(7):e103964. doi: 10.1371/journal.pone.0103964 (PMC4117593; doi:10.1371/journal.pone.0103964)
Supplement: Figure S1 — Distribution of Daphnia sampling sites in Michigan and Ontario. The five ponds are Disputed, Solomon and three ponds in the Canard area. The three lakes are Lawrence, Three Lakes II, and Warner. (PDF) [file pone.0103964.s001.pdf]

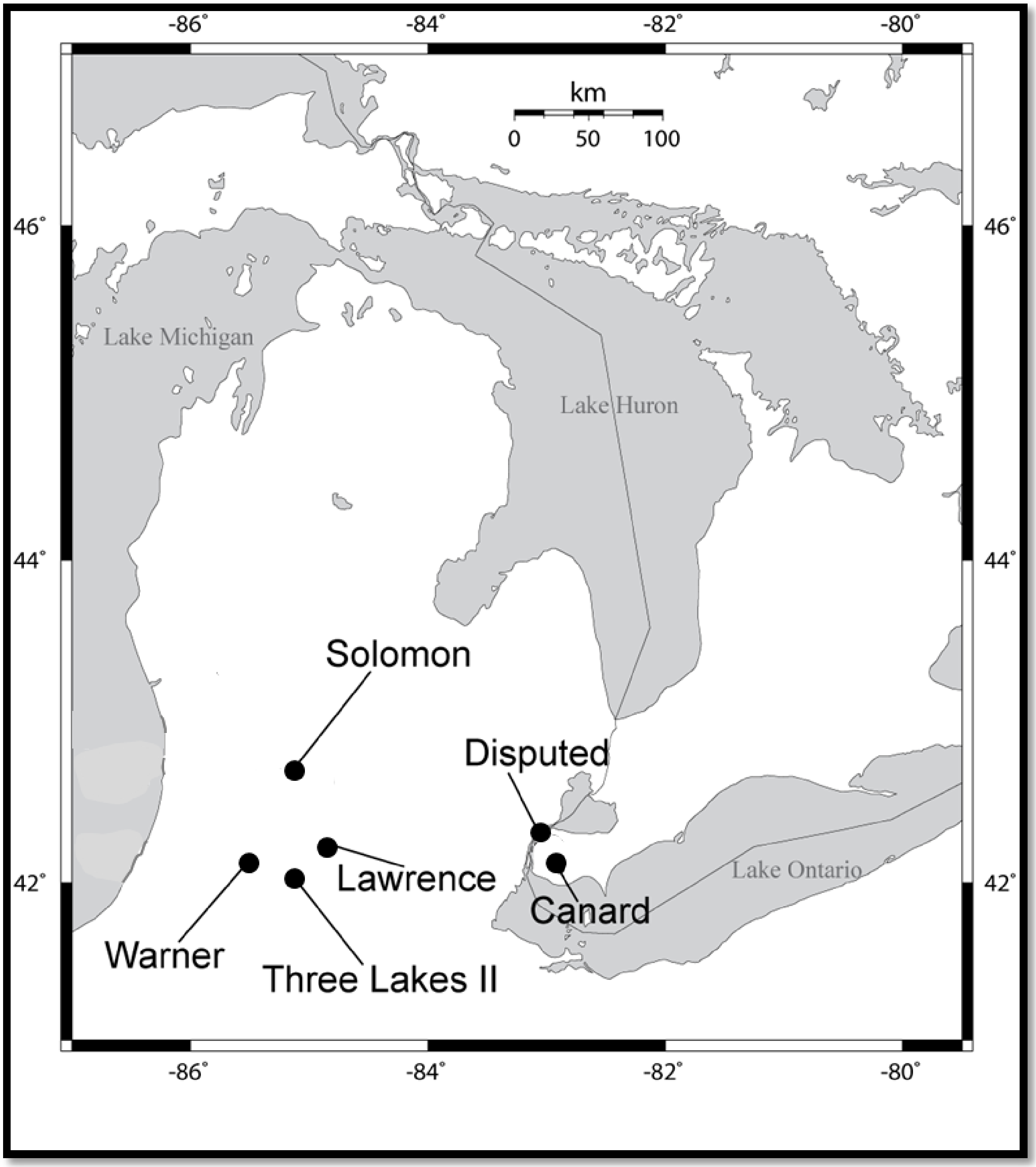

Additional File 1 - Distribution of *Daphnia* sampling sites in Michigan and Ontario.

The five ponds are Disputed, Solomon and three in the Canard area.

The three lakes are Lawrence, Three Lakes II, and Warner.
